# Vitamin D Antagonizes Negative Effects of Preeclampsia on Fetal Endothelial Colony Forming Cell Number and Function

**DOI:** 10.1371/journal.pone.0098990

**Published:** 2014-06-03

**Authors:** Frauke von Versen-Höynck, Lars Brodowski, Ralf Dechend, Ashley C. Myerski, Carl A. Hubel

**Affiliations:** 1 Department of Obstetrics and Gynecology, Gynecology Research Unit, Hannover Medical School, Hannover, Germany; 2 Experimental and Clinical Research Center (Max-Delbrück Center for Molecular Medicine and Medical Faculty of the Charité and Franz-Volhard Clinic), Helios Klinikum Berlin-Buch, Berlin, Germany; 3 Magee-Womens Research Institute and Department of Obstetrics, Gynecology & Reproductive Sciences, University of Pittsburgh School of Medicine, Pittsburgh, Pennsylvania, United States of America; Queen's University, Canada

## Abstract

**Context:**

Endothelial dysfunction is a primary feature of preeclampsia, a pregnancy complication associated with an increased future cardiovascular risk for mother and offspring. Endothelial colony forming cells (ECFC) are endothelial progenitor cells that participate in vasculogenesis and endothelial repair.

**Objective:**

We hypothesized that the number and functional properties of fetal cord blood-derived ECFCs are reduced in preeclampsia compared to uncomplicated pregnancy (controls), and asked if adverse effects of preeclampsia on ECFC function are reversed by 1,25 (OH)_2_ vitamin D_3_.

**Design, Setting, Patients:**

This was a nested, case-control study. Forty women with uncomplicated pregnancy and 33 women with PE were recruited at Magee-Womens Hospital (USA) or at Hannover Medical School (Germany).

**Main Outcome Measures:**

Time to ECFC colony appearance in culture, and number of colonies formed, were determined. Functional abilities of ECFCs were assessed *in vitro* by tubule formation in Matrigel assay, migration, and proliferation. ECFC function was tested in the presence or absence of 1,25 (OH)_2_ vitamin D_3_, and after vitamin D receptor (VDR) or VEGF signaling blockade.

**Results:**

The number of cord ECFC colonies was lower (P = 0.04) in preeclampsia compared to controls. ECFCs from preeclampsia showed reduced proliferation (P<0.0001), formed fewer tubules (P = 0.02), and migrated less (P = 0.049) than control. Vitamin D_3_ significantly improved preeclampsia ECFC functional properties. VDR- or VEGF blockade reduced tubule formation, partially restorable by vitamin D_3_.

**Conclusion:**

Fetal ECFCs from preeclamptic pregnancies are reduced in number and dysfunctional. Vitamin D_3_ had rescuing effects. This may have implications for the increased cardiovascular risk associated with preeclampsia.

## Introduction

Impaired placentation and maternal endothelial dysfunction are principal features of the pregnancy syndrome preeclampsia (PE) that affects 3–7% of all pregnancies [1], [2]. Effective preventive or therapeutic strategies do not exist to date [3]. PE has long-term, adverse health implications for both mother and offspring, including the development of hypertension and cardiovascular disease [4], [5]. However, the mechanisms linking an abnormal intrauterine environment to long-term endothelial dysfunction and vascular damage remain elusive.

Circulating endothelial progenitor cells (EPCs) are critical for blood vessel formation and repair [6]. EPC numbers and function inversely correlate with the risk of developing cardiovascular disease [7]. Based on these characteristics EPCs have been intensively studied in the context of cardiovascular risk [8]. Endothelial colony forming cells (ECFCs) are a well-defined subpopulation of EPCs. Unlike other EPC sub-types, they are directly involved in vasculogenesis and vascularization by populating the endothelial surface. They are involved in feto-placental vasculogenesis [9], which is disturbed in women with PE [10]. Although there is evidence that maternal and fetal (umbilical cord) circulating EPCs of hematopoietic lineage are reduced in number and function during PE [11], [12], [13], data on ECFCs are presently rare.

Vitamin D_3_ deficiency is associated with cardiovascular disease, hypertension, obesity, diabetes mellitus and metabolic syndrome [14], [15]. Compared with uncomplicated pregnancies, PE is characterized by marked changes in vitamin D_3_ and calcium metabolism [16]. A recent meta-analysis and several observational studies show a significant relationship between vitamin D deficiency and an increased risk for PE [17], [18], [19]. Moreover, PE is associated with a reduced placental and fetal vitamin D pool [20]. We recently showed a significant promotion of *in vitro* angiogenesis by 1,25 (OH)_2_ vitamin D_3_ in fetal ECFCs, related to an increase in VEGF expression and pro-MMP-2 activity, suggesting a regulatory role of vitamin D for ECFC function [21].

We hypothesized that cord blood ECFC number/abundance and *in vitro* proliferative and vasculogenic capacity would be reduced in PE compared to uncomplicated pregnancies. We further sought to determine whether the ECFC angiogenesis-related functional differences can be neutralized by vitamin D. We compared the number of ECFC outgrowth colonies arising in culture according to outcome group. We also compared functional attributes of PE and uncomplicated pregnancy ECFCs in culture, namely tubule-like structure formation in Matrigel assay, migration and proliferation, in the presence and absence of supplemental vitamin D. Further, we tested effects of vitamin D receptor (VDR) and vascular endothelial growth factor (VEGF) receptor protein tyrosine kinase 1/2 blockers on tubule formation capacity of PE and uncomplicated pregnancy ECFCs in the presence and absence of vitamin D.

## Materials and Methods

### Patients

This was a collaborative study by members of Magee-Womens Research Institute (MWRI) and Hannover Medical School (MHH). The University of Pittsburgh Institutional Review Board and the Ethical Committee at MHH approved the study. Informed written consent was obtained from each patient. ECFCs were isolated from cord blood of 40 uncomplicated (MWRI: 36; MHH: 4) and 33 PE pregnancies (MWRI: 30; MHH: 3) delivered by vaginal or Cesarean section (Tables S1 and S2).

PE was diagnosed by the presence of gestational hypertension and proteinuria beginning after the 20^th^ week of pregnancy, with resolution of clinical symptoms postpartum. Gestational hypertension was defined as persistent, new onset hypertension (absolute blood pressure ≥140 mmHg systolic and/or ≥90 mmHg diastolic) appearing after 20 weeks of gestation [22]. Proteinuria was defined as ≥300 mg per 24-h urine collection, ≥2+ protein on voided urine sample, ≥1+ protein on catheterized urine specimen, or a protein-creatinine ratio of ≥0.3. The study subjects were classified as having an uncomplicated pregnancy if they were normotensive and without proteinuria throughout gestation, and if they delivered healthy babies. All women had singleton pregnancies. All patients had no clinical history of preexisting diabetes or renal, hypertensive or vascular disease, and did not use illicit drugs.

Pre-pregnancy weight, self-reported at enrollment, and measured height were used to calculate pre-pregnancy body mass index (BMI; weight [kg]/height [m^2^]). Maternal race was by self-report at enrollment. Self-report, during pregnancy or immediately postpartum, was used to collect data on tobacco smoking (y/n). Gestational age-specific birth weight percentiles, adjusted for infant sex and race, were based upon data from Magee-Womens Hospital (Pittsburgh, Pennsylvania) or Hannover Medical Center (Hannover, Germany).

### ECFC isolation and culture

ECFCs from cord blood were isolated as previously described [21]. Briefly, umbilical cord venous blood (15–20 ml) was collected immediately after delivery into sterile EDTA-coated tubes. Blood samples were centrifuged within 3 h of collection at 2,000 g for 20 min. Peripheral blood mononuclear cells (PBMCs) were isolated by density gradient centrifugation. The plasma was removed for collection and replaced with the same volume of plasma replacement buffer consisting of phosphate buffered saline solution (PBS) supplemented with 0.025 M EDTA (Sigma Aldrich, Steinheim, Germany or St. Louis, MO) and 1% (v/v) penicillin/streptomycin (Sigma Aldrich). The sample volume was doubled by adding isolation buffer (PBS, 2% (v/v) fetal bovine serum [FBS, Biochrom KG, Berlin, Germany or Life Technologies, Carlsbad, CA], 1% penicillin/streptomycin), and the sample was gently mixed. Samples were layered on Ficoll Plus (GE Healthcare, Buckinghamshire, England or Piscataway, NJ) and spun at 400 g for 40 min in a swinging bucket centrifuge with brake in the off position. The PBMC fraction was collected and washed two times with isolation buffer. Peripheral blood mononuclear cells were cultured in endothelial growth medium 2 (EGM-2, Lonza, Basel, Switzerland or Walkersville, MD), supplemented with supplier-recommended concentrations of human recombinant epidermal growth factor, VEGF, ascorbic acid, hydrocortisone, heparin and recombinant insulin-like growth factor, 10% FBS and 1% penicillin/streptomycin. The PBMCs were plated at a density of 5×10^7^ cells/well on collagen coated 6-well plates (BD Bioscience, Heidelberg, Germany or Billerica, MA) and incubated at 37°C, 5% CO_2_. Medium was changed daily for 10 days and then every second day. First appearance of ECFC colonies was noted as well-circumscribed monolayers of >50 rapidly proliferating, cobblestone-appearing cells. Colonies were identified by visual inspection using an inverted microscope (Olympus, Tokyo, Japan; Zeiss, Thornwood, NY). Well- defined colonies were released from the plates using cloning rings and trypsin-EDTA and collected. The cells from each separate colony were placed into a well of a collagen-coated 6 well plate and after becoming 80–90% confluent, subsequently passaged into collagen-coated T25 culture flasks. After becoming 80–90% confluent, the cells in the T25 flasks were passaged into gelatin-coated T75s. At 80–90% confluence these cells were harvested and frozen in freezing medium containing 92% FCS and 8% DMSO (Sigma Aldrich, Steinheim, Germany). All experiments were run with ECFCs in passage 5. ECFCs obtained from cryovials were re-thawed within 3 min in warm water bath and seeded into 75 cm^2^ tissue culture flasks and used at 80–90% confluence. For each functional experiment, ECFCs from PE and a corresponding control patient were run in tandem.

### ECFC Characterization

ECFCs were characterized by immunophenotyping and flow cytometry as described previously [21]. To assess the ability of cells to take up Dil-acetylated-low-density lipoprotein (Dil-Ac-LDL; Biomedical Technologies, Stroughton, MA) and fluorescein isothiocyanate-labeled Ulex europaeus agglutinin I (lectin; Sigma-Aldrich, Steinheim, Germany) attached cells were incubated with 5 µg/ml Dil-Ac-LDL and incubated for 4 h at 37°C and permeabilized with Tergitol-type NP-40 for 1 min. Cells were fixed with 4% paraformaldehyde for 10 min and counterstained with 10 µg/ml lectin for 1 h. DAPI (Thermo Scientific, Rockford, IL) was used for staining nuclei. Double-positive fluorescence cells for Dil-Ac-LDL (456 nm) and lectin (488 nm) were identified as differentiating ECFCs using a Leica EL600 fluorescence microscope (Leica Microsystems, Wetzlar, Germany). Flow cytometric analyses to confirm the ECFCs phenotype were performed using surface markers CD31, CD34, CD133, VEGFR-2, and CD45 as well as appropriate isotype controls. Using 5 mM EDTA (in PBS) cultivated ECFCs were detached from culture plasticware and 0.5×10^6^ cells were used per FACS tube and solubilized in FACS buffer (0.1% BSA and 0.02% sodium azide in DPBS) before Fc-receptor blocking reagent (Miltenyi, Auburn, CA) was added. Isotypes or antibodies were added to the preparations, cells got washed and were analyzed using the BD flow cytometer LSR-II and Software BD FACSDiva.

### VDR silencing

For silencing of the vitamin D receptor (VDR) ECFCs were transiently transfected with specific VDR small interfering (si) RNA (VDR silencer validated siRNA, Ambion, AM51331, Life Technologies, Darmstadt, Germany, 50 nM) or negative siRNA (Life Technologies, Carlsbad, USA), diluted in EGM +10% (v/v) FCS without antibiotics and containing Dharmafect 1 transfection reagent (Dharmacon, Darmstadt, Germany). siRNA transfection solution was added to ECFCs in a 6-well plate at 80–90% confluence. After 24 h of incubation the media was replaced with regular growth medium (EGM-2) and cells were used for further experiments. Efficiency of transfection was visualized by fluorescence staining and the VDR expression was tested by real-time RT-PCR. We confirmed a mean reduction of 64±0.14% after silencing of the VDR compared to untreated control cells, and no significant difference between non-targeting siRNA and untreated control (Figure S1).

### *In vitro* angiogenesis assay

The capacity of ECFCs to form capillary tubule-like networks was tested by seeding 17,000 cells/well in 96-well plates pre-coated with 50 µL growth factor reduced Matrigel (BD Biosciences, Bedford, MA). The cells were incubated for 14 h, with 0 nM (vehicle), 1 nM or 10 nM of 1,25 (OH)_2_ vitamin D_3_ (Sigma Aldrich, St. Louis, MO), in endothelial basal medium (EBM; without supplements) containing 0% or 5% v/v FBS. Light microscopy images were obtained at 2.5× magnification. The concentrations of 1,25 (OH)_2_ vitamin D_3_ were intended to approximate physiological levels in pregnancy [23], [24]. Total tubule length in each visual field was measured using ImageJ freeware (NIH Image). Each treatment was done in triplicate wells. Triplicate data were averaged, and with experimental “n” corresponding to the patient sample evaluated.

### Proliferation assay

To determine the proliferative capacity of ECFCs derived from uncomplicated and PE pregnancies in the presence or absence of 1,25 (OH)_2_ vitamin D_3_, 10,000 cells were seeded per well of 24-well culture plates in EGM supplemented with 8% (v/v) FBS and 1% penicillin/streptomycin. Medium was changed the next day and cells were incubated with 0 nM (vehicle), 1 nM or 10 nM of 1,25 (OH)_2_ vitamin D_3_. After 24 h, 48 h and 72 h of treatment, the cell number was counted in a Neubauer chamber with 1∶2 trypan-blue dilution. Population doubling time was calculated as following: log2/ (logNt – logNo), t =  time period (h), Nt =  number of cells at time t, No  =  initial cell number.

### Migration assay

Cell migration was assessed using a “scratch wound healing assay” [25]. 80,000 ECFCs/well were seeded on gelatin coated wells of 24-well culture plates with EGM containing 10% FBS. When cells reached 70–80% confluence the medium was changed to EBM (without growth supplements) with 2% FBS and cells were pre-incubated with 0 nM (vehicle), 1 nM or 10 nM 1,25 (OH)_2_ vitamin D_3_ for 24 h. The cell monolayers were then scratched with a sterile P200 pipette tip. The media was aspirated and replaced with the same media to remove non-adherent cells. Light microscopic images were immediately obtained (incubation start time, 0) and obtained again after 8 h. Non-populated scratch areas were quantified by ImageJ software, and the percent closure (percentage of original area occupied at 8 h by cells that had migrated into the wound area) was calculated. All experiments were done in quadruplicate wells.

### VDR blocking and VEGF pathway inhibition

To test the VDR receptor-dependency of functional effects of 1,25 (OH)_2_ vitamin D_3_, we silenced the VDR with siRNA, or blocked the VDR with the VDR antagonist pyridoxal-5-phosphate (0.5 mM, Cell Signaling/New England Biolabs, Frankfurt am Main, Germany) as described previously [21]. We tested effects of the Flk-1/KDR (VEGF) receptor tyrosine kinase inhibitor SU5416 (0.5 µM, Sigma Aldrich, Steinheim, Germany). *In vitro* Matrigel tube formation assays were performed (as described above) in presence or absence of 1,25 (OH)_2_ vitamin D_3_ (10 nM) and with or without the VDR or VEGF inhibitor or after silencing of the VDR.

### Statistical analysis

Demographic data are expressed as means and standard deviation. Experimental data are presented as means and standard error. Distribution was examined with Kolmogorov- Smirnov test. Continuous data were compared with ANOVA, Kruskal-Wallis, unpaired t-test, Mann-Whitney or Wilcoxon-signed rank test, as appropriate. Categorical variables were compared by Fisher's exact test. Data were analyzed with Prism 4 software package (GraphPad Software Inc., La Jolla, CA). Where specified, to account for interassay variation, data are given as fold change in functional variables relative to untreated ECFCs from uncomplicated pregnancies or relative to untreated ECFCs from PE pregnancies.

## Results

### Patients demographics

The clinical and demographic data for the pregnant women who provided blood samples for the analysis of ECFC abundance and colony formation are presented in Table S1. Data describing the sub-set of patients whose ECFCs were functionally compared, are given in Table S2. Maternal age, maternal pre-pregnancy body-mass index, race and parity, and baby gender were not statistically different between the PE and control groups (Tables S1 and S2). The percentage of women who were delivered by Cesarean section versus vaginal delivery did not differ by outcome group. By definition, women with PE had significantly higher systolic and diastolic blood pressures at delivery compared to the uncomplicated study group. Patients of the two groups described in Table S2 were matched by gestational age for the cell culture experiments. Infant birth weights and birth weight percentiles were lower in PE compared to controls.

### Morphology and Characterization of ECFCs

ECFC emerged in culture as discrete, late outgrowth colonies displaying the characteristic cobblestone morphology. Dil-Ac-LDL uptake and lectin binding were observed in these cells, consistent with an endothelial phenotype. In addition flow cytometric results showed that ECFCs through passage 10 were homogenous and had the typical phenotype of endothelial cells, being CD31+, CD45-, and CD133-. The expression of CD34 decreased with increasing culture time becoming negative at passage 10–15 as described by Fina et al. [26] (data not shown).

### Cord blood ECFC colony numbers and time of first appearance

The number of cord blood ECFC colonies was significantly decreased in PE compared with controls (mean ±SD number of colonies per 5×10^7^ PBMCs: 2.2±3.6 (control) vs. 1.0±1.8 (PE); P = 0.04 by Mann-Whitney test). Twenty-four of 36 (67%) control cord blood samples yielded one or more colonies, whereas colonies manifested from 13 of 30 (43%) of PE samples (P = 0.06). There was no difference in the number of days until first appearance of colonies (>50 characteristic cells), (mean ±SD days: 16.9±5.6 (control) vs. 17.5±4.2 (PE); p = 0.73). In addition there was no significant correlation of birth weight percentile and time to appearance of colonies (r = −0.01, P = 0.57 (control); r = −0.19, P = 0.31 (PE)). The demographic data of the patients are depicted in Table S1.

### *In vitro* angiogenesis

**Figure 1:**
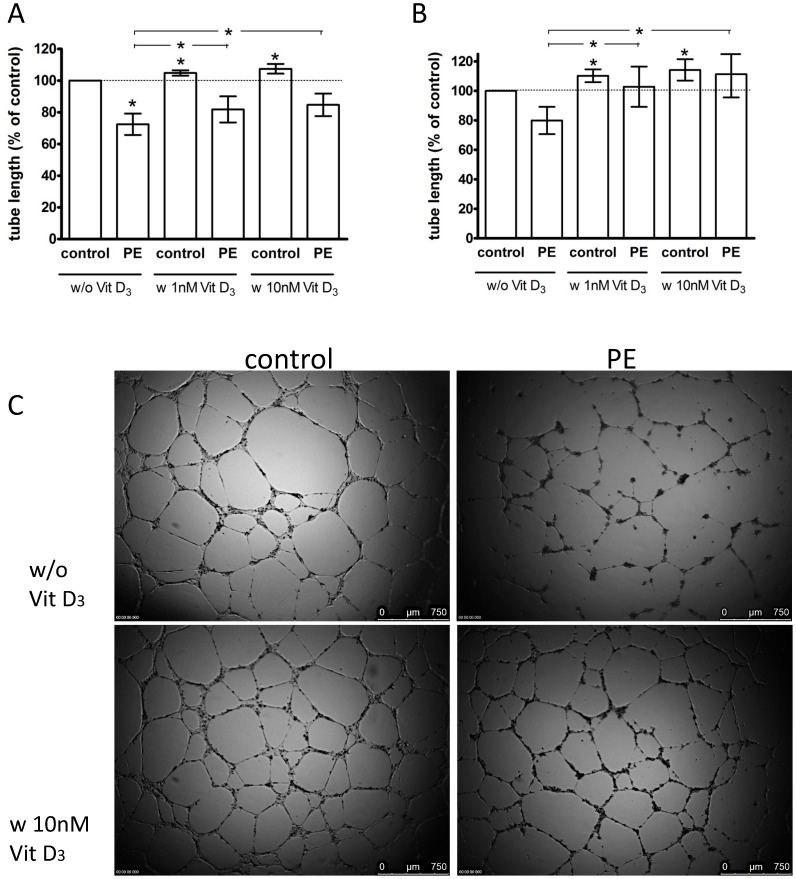
Effect of preeclampsia (PE) and 1,25(OH)_2_ vitamin D_3_ on capillary-tube formation in a Matrigel assay. ECFCs were cultured in endothelial basal medium (EBM) + 5% FBS (A) or without FBS (B) and treated with 1 nM or 10 nM 1,25(OH)_2_ vitamin D_3_ or without vitamin D_3_. Control: uncomplicated pregnancy. Capillary-tube formation (average total tubule length per microscopic field) was analyzed after 14 h by visual microscopy at 2.5× magnification. Data are expressed as percentage of control. Results represent mean values of total tubule length ±SEM of at least 8 independent experiments. *P<0.05 vs. untreated control or (as indicated by horizontal lines above the vertical bars) vs. untreated PE for 1,25(OH)_2_ vitamin D_3_ effects. C: Representative photomicrographs of ECFCs in Matrigel assay with EBM +5% FBS. Scale bar represents 750 µm.

A Matrigel model was used to assess the capacity of ECFCs to differentiate into tubule-like structures. Figure 1 displays total tubule lengths per microscopic field, all as percent relative to untreated, uncomplicated pregnancy ECFCs (control, 100%). Tubule assemblage by PE-derived ECFCs was markedly impaired (72±7, p = 0.02, unpaired t test) (Figure 1A). The experiments represented in Figure 1A were performed in the presence of 5% (v/v) FBS; similar results were obtained in the absence of FBS, although no significant differences were reached (Figure 1B).

The impact of 1,25(OH)_2_ vitamin D_3_ on capillary-tube formation of control and PE ECFCs was evaluated after 14 h of incubation, in the absence or presence of 5% FBS and 1,25(OH)_2_ vitamin D_3_. Under incubation with 5% FBS, supplementation with 1 nM (105±2, P = 0.01) or 10 nM (108 ± 3, P = 0.04) 1,25(OH)_2_ vitamin D_3_ resulted in a small but significant increase of tubules formed by uncomplicated pregnancy ECFCs (data expressed as percent relative to vitamin D-untreated ECFCs). A significant stimulatory effect of vitamin D_3_ was also observed in the absence of FBS, by 1 nM (110±4, P = 0.04) and 10 nM (114±7, P = 0.02) 1,25(OH)_2_ vitamin D_3_, respectively. When expressed as percent relative to vitamin D-untreated PE ECFCs (100%), in 5% FBS, both 1 nM (113±3, P = 0.001) and 10 nM (119±7, P = 0.02) of 1,25(OH)_2_ vitamin D_3_ stimulated the formation of tubules by ECFCs from women with PE. This effect of vitamin D was more pronounced in culture medium without FBS: 1 nM (127±6, P = 0.004) or 10 nM (137±8, P = 0.003) 1,25(OH)_2_ vitamin D_3_. Consequently, both 1 nM (5% FBS: P = 0.08; 0% FBS: P = 0.4) and 10 nM (5% FBS: P = 0.1; 0% FBS: P = 0.6) 1,25(OH)_2_ vitamin D_3_ neutralized the PE vs. uncomplicated pregnancy differences in the capacity of ECFCs to form tubules.

### Migration

**Figure 2:**
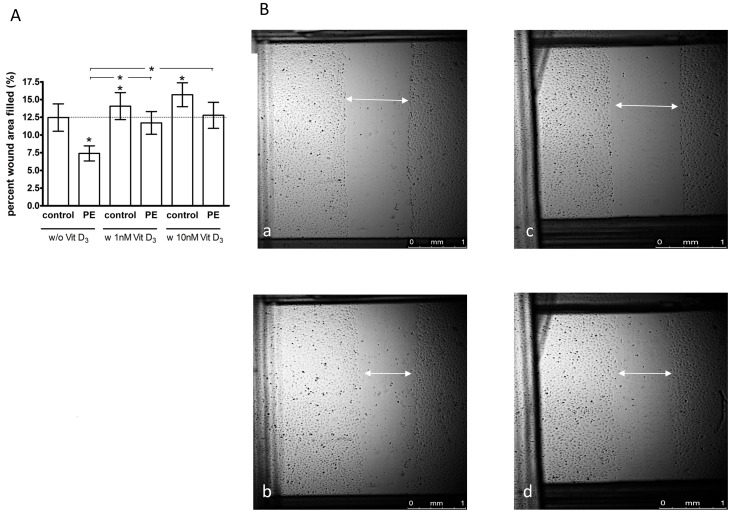
Effect of preeclampsia (PE) and 1,25(OH)_2_ vitamin D_3_ on ECFC migration. ECFCs of uncomplicated (control) and PE pregnancies were cultured in endothelial basal medium (EBM) +2% FBS and treated with or without 1 nM or 10 nM 1,25(OH)_2_ vitamin D_3_. A: The migration of ECFCs into the scratch wound was assessed after incubation for 8 h. Results represent mean ±SEM percent wound filling, n = 8. *P<0.05 vs. untreated control or (as indicated by horizontal lines above the vertical bars) vs. untreated PE. B: Representative images of ECFC monolayers with scratch wounds at 0 h (a, c) and 8 h (b, d) of incubation of control (a, b) and preeclamptic pregnancies (c, d).

With scratch wound area filling expressed as percent relative to untreated, uncomplicated pregnancy ECFCs (control, 100%), the migration of PE ECFCs was significantly impaired (66±9, P = 0.04), (Figure 2 A, B). 1,25(OH)_2_ vitamin D3 improved ECFC migration in both pregnancy outcome groups. At 1 nM (118±8, P = 0.04) or 10 nM (142±24, P = 0.02) the migration of uncomplicated pregnancy ECFCs was significantly stimulated. Expressed as percent relative to untreated PE (100%), migration into the scratch wound of PE ECFCs was also significantly higher after treatment with 1 nM (163±16, P = 0.01) and 10 nM (188±37, P = 0.02) 1,25(OH)_2_ vitamin D3. Therefore, vitamin D restored ECFC migration derived from PE pregnancies such that there was no significant between-group difference after treatment with 1 nM (P = 0.4) or 10 nM (P = 0.3) 1,25(OH)_2_ vitamin D_3._

### Proliferation

**Figure 3:**
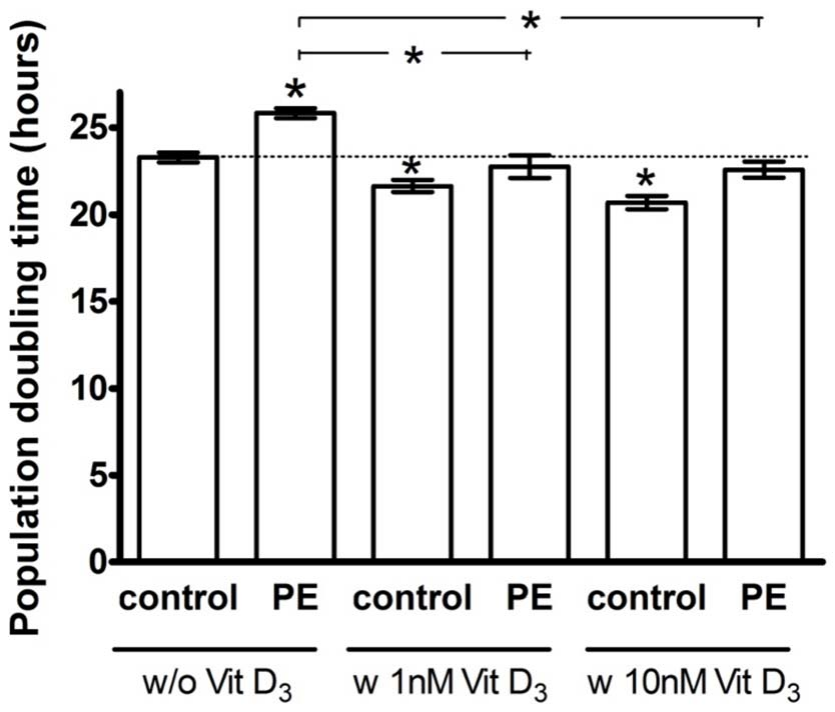
Effect of pregnancy outcome and 1,25(OH)_2_ vitamin D_3_ on ECFC population doubling time. ECFCs of uncomplicated (control) and preeclamptic (PE) pregnancies were incubated in the presence and absence of 1,25(OH)_2_ vitamin D_3_ (1 nM or 10 nM) in EGM +8% (v/v) FBS. Cell numbers were counted and population doubling time calculated after 72 h. Population doubling time was significantly longer in PE ECFCs compared to uncomplicated pregnancy (control) in the absence of supplemental vitamin D (P<0.05). PE population doubling time was reduced to control levels by vitamin D, n = 8. *P<0.05 vs. untreated control or (as indicated by horizontal lines above the vertical bars) vs. untreated PE for 1,25(OH)_2_ vitamin D_3_ effects.

Figure 3 shows the population doubling time of ECFCs, all expressed relative to untreated, uncomplicated pregnancy ECFCs (100%). Population doubling time of ECFCs from PE pregnancies was significantly prolonged compared to uncomplicated pregnancy ECFCs during 72 h of culture (111±2, P<0.0001). Concentrations of 1 nM (93±1, P<0.001) or 10 nM (89±1, P<0.0001) 1,25(OH)_2_ vitamin D_3_ significantly reduced the doubling time of uncomplicated pregnancy ECFCs. With data expressed as percent relative to vitamin D-untreated PE ECFCs (100%), both the 1 nM (88±2, P = 0.002) and 10 nM (88±2, P = 0.02) 1,25(OH)_2_ vitamin D3 treatments significantly shortened the population doubling time of PE ECFCs. Group differences in ECFC proliferation were neutralized by 1 nM 1,25(OH)_2_ vitamin D3 (P = 0.07). Although 10 nM 1,25(OH)_2_ vitamin D3 reduced the population doubling time in both groups, the doubling time remained shorter in controls (P = 0.01).

### VDR blocking and inhibition of VEGF pathway

**Figure 4:**
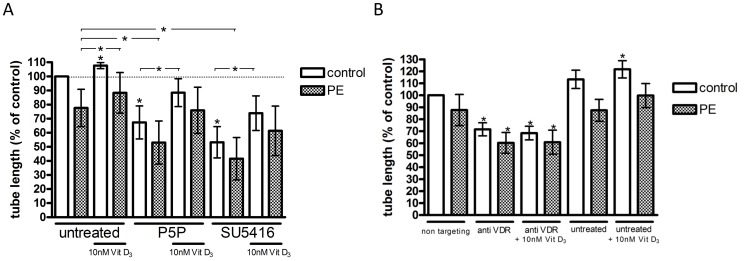
Effect of 1,25(OH)_2_ vitamin D_3_ and the inhibitors pyridoxal-5-phosphate, SU5416 and vitamin D receptor (VDR) small interfering (si)RNA on capillary-tube formation. ECFCs of uncomplicated (control) and preeclamptic (PE) pregnancies were cultured in EBM +5% (v/v) FBS and treated with 10 nM 1,25(OH)_2_ vitamin D_3_ for 14 h. Control: uncomplicated pregnancy. PE: pregnancy complicated by preeclampsia. Capillary-tube formation was determined by seeding 17,000 ECFCs on 50 µl Matrigel and incubated with 0.5 mM pyridoxal-5-phophate, 0.5 µM SU5416 (A) or 50 nM VDR siRNA or non-targeting siRNA (B) each either without additional 1,25(OH)_2_ vitamin D_3_ or with 10 nM 1,25(OH)_2_ vitamin D_3_ treatment respectively. Tubule length was analyzed after 14 h by visual microscopy at 2.5× magnification. Results represent mean ±SEM of at least 4 independent experiments. *P<0.05 compared to control.

We silenced the VDR with siRNA or blocked the VDR with the VDR antagonist pyridoxal-5-phosphate (P5P). To test whether VEGF signaling could be involved, we pre-treated cells with the Flk-1/KDR (VEGF) receptor tyrosine kinase inhibitor SU5416. Tubule lengths per microscopic field in Figure 4 are expressed as percentage relative to values from untreated, uncomplicated pregnancy ECFCs, with experiments performed in the presence of 5% (v/v) FBS. As shown in Figure 4 A, tubule formation by PE ECFCs was lower than uncomplicated pregnancy ECFCs in the absence of vitamin D (76±13, P = 0.08, n = 4). Treatment with 10 nM vitamin D increased tubule formation in both groups (uncomplicated pregnancy: 108±2, P = 0.03, n = 5; PE: 114±3, P = 0.02, n = 4, (data expressed as percent relative to vitamin D-untreated uncomplicated pregnancy or PE ECFCs, respectively).

In the absence of vitamin D, treatment with P5P impaired angiogenesis in both uncomplicated pregnancy (67±11, P = 0.048, n = 5) and PE ECFCs (64±10, P = 0.04, n = 4), as did the VEGF pathway inhibitor SU5416 (uncomplicated pregnancy: 53±11, P = 0.01, n = 5; PE: 48±12, P = 0.02, n = 4). In the presence of 10 nM 1,25(OH)_2_ vitamin D3, the inhibiting effects of P5P on ECFC tubule formation remained significant but were less pronounced (uncomplicated pregnancy: 88±10, P = 0.04, n = 5; PE: 95±5, P>0.05, n = 4). In the presence of 10 nM 1,25(OH)_2_ vitamin D3 the inhibitory effects of SU5416 were also partially reversed (uncomplicated pregnancy: 74±12, P = 0.01, n = 5; PE: 75±14, P>0.05, n = 4).

VDR knock down by siRNA had a significant suppressive effect on tube formation of PE ECFCs (69±3, P = 0.003, n = 4) compared to non-targeting (scrambled) siRNA. VDR siRNA also caused a significant inhibitory effect on tubule formation of uncomplicated pregnancy ECFCs (72±5, P = 0.007, n = 5) compared to non-targeting siRNA. Vitamin D had no effects on tubule formation after siRNA treatment (Figure 4 B). Tubule formation did not differ between non-targeting siRNA and no siRNA treatments (vehicle), (Figure 4B).

## Discussion

Here we have shown differences in number and function of fetal ECFCs isolated from cord blood of uncomplicated pregnancies compared to PE pregnancies. The number of ECFC colonies obtained from PE pregnancies was significantly lower, and functional properties, i.e. proliferation, migration and tubule formation, were impaired compared to ECFCs from healthy pregnancies. These effects were largely independent of the FBS concentration used in the experiments. In the presence of 1,25 (OH)_2_ vitamin D_3_, the functional properties improved and the negative effects of preeclampsia on fetal ECFCs were significantly lessened. The positive effects of vitamin D were dependent on VDR activation, as indicated by experiments with either VDR gene silencing (siRNA) or VDR blockade (pyridoxal-5-phosphate). The findings suggest that ECFC tubule formation in the presence of FBS is, at least in part, VDR dependent. The vitamin D pathway appeared to involve stimulation of the VEGF signaling cascade, given that SU5416 suppressed ECFC tubule formation in a fashion similar to VDR blockade. Except for siRNA knockdown of the VDR these effects were substantially reversed by a co-treatment with 1,25 (OH)_2_ vitamin D_3_. We speculate that a displacement in form of a competitive antagonism by vitamin D at the receptor reduced the negative effects of pyridoxal-5-phosphate and SU5416 on angiogenesis. The reason why inhibition of VDR, either through pharmacological intervention or siRNA, reduced tube lengths in the absence of supplemental vitamin D is unknown. It is possible that vitamin D in fetal bovine serum (FBS) is sufficient to promote submaximal tubule formation. In our earlier publication we likewise observed a reduction of ECFC tubule formation in Matrigel upon inhibition of the VDR with siRNA in the absence of supplemental vitamin D [21]. In that study we surprisingly observed that 10 nM 1,25(OH)_2_ vitamin D in the presence of VDR siRNA caused a further reduction in tubule formation. We speculated that the higher levels of vitamin D might exert inhibitory effects by activating a membrane bound (non-classical) VDR, when the nuclear VDR is downregulated.

Our findings confirm data of our previous study in which we demonstrated a stimulating effect of 1,25 (OH)_2_ vitamin D_3_ on fetal ECFC function in uncomplicated pregnancies [21]. To our knowledge, however, this is the first study to demonstrate functional deficits of fetal ECFC from pregnancies complicated by PE compared to uncomplicated pregnancies, and significant restoration of function by vitamin D.

Endothelial colony forming cells (ECFC) are a subset of endothelial progenitor cells and critical to blood vessel formation and repair [6]. Their dysfunction represents a risk factor for cardiovascular disease [27]. Previous studies of endothelial progenitor cells with hematopoietic (non-ECFC) characteristics (CD133^+^ and/or CD45^+^) found lower circulating numbers and reduced colony-forming ability in PE compared to control maternal blood samples [12], [13]. This implicates a source of maternal endothelial dysfunction by lessening endothelial repair and vasculogenic capacity. Due to their rarity in the maternal compared to fetal circulation, impractically large volumes of maternal blood (≥50 mL) would likely be necessary to obtain sufficient numbers of maternal blood ECFCs for study [28].

Our unpublished data indicate no effect of 1 or 10 nM concentrations of 1,25(OH)_2_ vitamin D on tube formation, migration or proliferation of human umbilical vein endothelial cells (HUVEC) in culture. This, combined with several reports that 1,25(OH)_2_ vitamin D either decreases or has no effect on endothelial cell proliferation or angiogenesis *in vivo* or *in vitro* [29], [30], might reflect heterogeneity among endothelial cell subtypes. ECFCs reportedly differ from HUVEC or human umbilical artery endothelial cells in the expression of differentiation-related surface markers (such as CD44) or activities such as proliferation or telomerase activities [31]. However the reason for the distinct proangiogenic response to vitamin D by ECFCs is presently unclear. Circulating hematopoietic endothelial progenitor cells, a more abundant progenitor type compared to ECFCs, respond to vitamin D and express functional vitamin D receptors. Interestingly, conditioned media from vitamin D (10 nM) treated hematopoietic progenitors increased tubule networks of human aortic endothelial cells in the Matrigel angiogenesis assay, whereas vitamin D alone did not [32]. To speculate, vitamin D might upregulate release of angiogenic factors by ECFCs that stimulate in an autocrine fashion.

Our data are similar in pattern to previous studies that showed a significantly decreased number of fetal hematopoietic EPCs from PE compared with those of normal pregnancy [33], [34], [35]. Moreover, fetal hematopoietic EPCs from preeclamptic women were more senescent and free VEGF was significantly decreased compared to controls [33], [34]. However, the progenitor cells in the aforementioned publications fail to form vessels *in vivo* or *in vitro*, and are clonally distinct from ECFCs [36].

Reduced fetal ECFC numbers and colony formation have also been observed in diabetic or growth restricted (IUGR) pregnancies, suggesting potential mechanistic insights into the long-term cardiovascular complications observed in newborns [37], [38], [39]. *In vivo*, IUGR ECFCs formed fewer blood vessels and capillaries compared to normal pregnancy-derived ECFCs. In culture, IUGR ECFCs showed reduced proliferation and migration abilities combined with a reduced hypoxia-induced MMP-2 release [38]. However, we found no significant correlation between birth weight percentile and ECFC variables in either group (data not shown). Furthermore, when excluding the SGA pregnancies, the results for tubule formation and population doubling time remained significant despite the reduced sample size (data not shown). This suggests that group differences were primarily driven by PE.

EPCs remain difficult to define due to the lack of a specific and unique cell surface or molecular marker. The ECFCs used in our study were rigorously identified using a combination of cell surface markers (CD34^+^, CD133^−^, CD45^−^), immunostaining and morphologic measures, e.g. colony-forming ability. Future comparison of ECFC function and effects of vitamin D in vasculogenic assays *in vivo* (e.g. mouse Matrigel plug assay [38]) would be useful.

In summary, this is to our knowledge the first report demonstrating fetal ECFC dysfunction in preeclampsia, and a marked improvement of fetal ECFC function with neutralization of negative effects of preeclamptic pregnancy by 1,25 (OH)_2_ vitamin D_3_. There appear to be adverse cardiovascular health implications, including increased risk of stroke, for adult offspring of preeclamptic pregnancies [40]. A recent systematic review concluded that young offspring of preeclamptic pregnancies already have increased blood pressure and BMI, both risk factors for future cardiovascular disease [41]. However, currently the mechanisms underlying these associations remain unclear and preventive strategies do not exist. It is plausible that avoidance of hypovitaminosis D could contribute to a reduction in feto-placental endothelial dysfunction, reduction in adverse pregnancy outcomes, and possibly have long-term implications for the cardiovascular health of offspring.

## Supporting Information

Figure S1 **Effect of VDR silencing on VDR gene expression.** ECFCs were transiently transfected with specific VDR small interfering (si) RNA (50 nM) or non-targeting siRNA for 24 h. VDR gene expression was tested by real-time RT-PCR. Results represent mean ±SEM of 6 independent experiments. *P<0.05 compared to control.
(TIF)

Table S1 **Clinical and demographic data for the uncomplicated and preeclamptic pregnancy patients, from whom blood samples were obtained.**
(DOCX)

Table S2 **Clinical and demographic data describing the sub-set of patients whose ECFCs were functionally compared.**
(DOCX)
