# Supplementary figures and images for: Vitamin D Antagonizes Negative Effects of Preeclampsia on Fetal Endothelial Colony Forming Cell Number and Function
Source: PLoS One. 2014 Jun 3;9(6):e98990. doi: 10.1371/journal.pone.0098990 (PMC4044051; doi:10.1371/journal.pone.0098990)

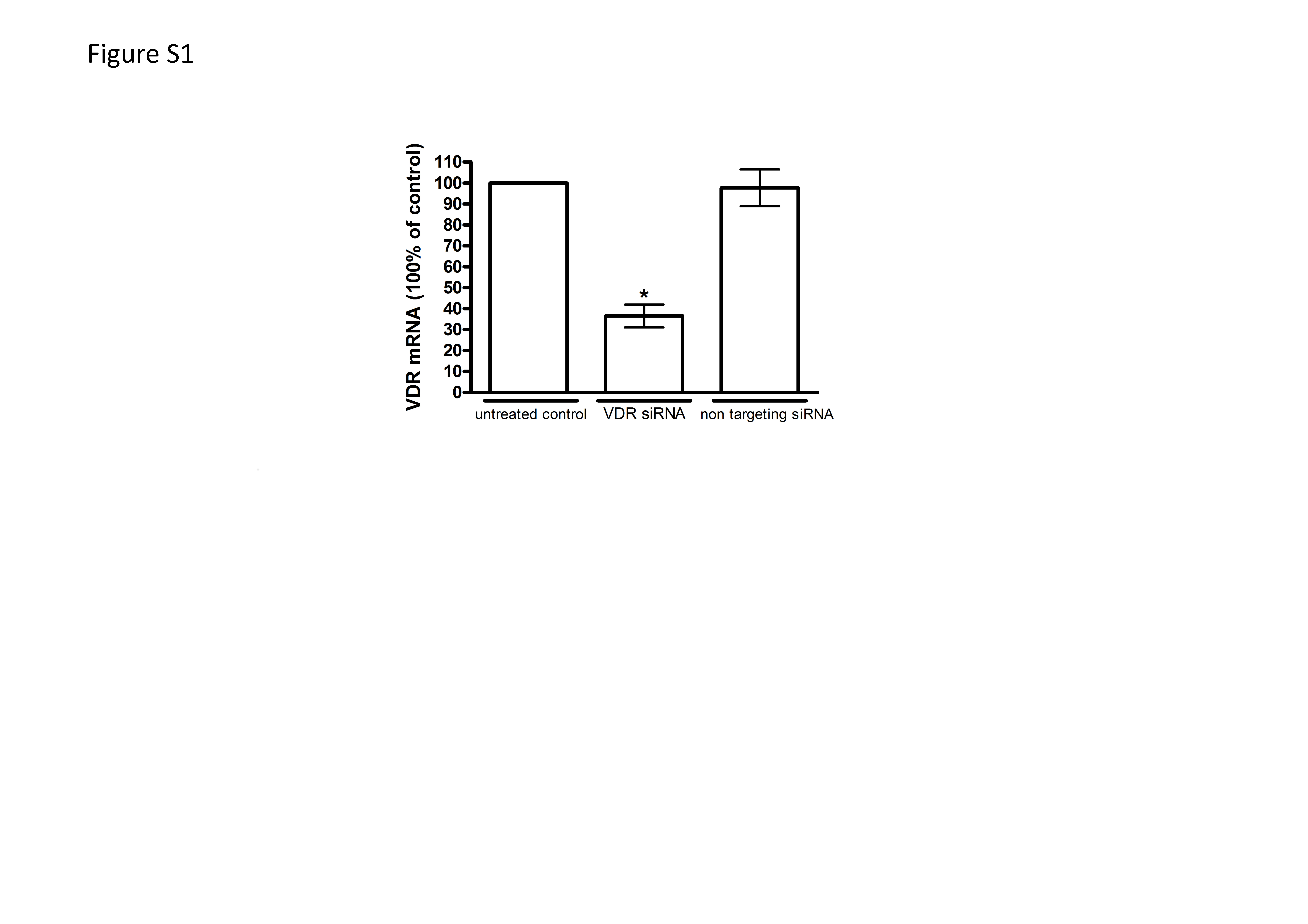

Supplement: Figure S1 — Effect of VDR silencing on VDR gene expression. ECFCs were transiently transfected with specific VDR small interfering (si) RNA (50 nM) or non-targeting siRNA for 24 h. VDR gene expression was tested by real-time RT-PCR. Results represent mean ±SEM of 6 independent experiments. *P<0.05 compared to control. (TIF) [file pone.0098990.s001.tif]
