# Supplementary material for: Vitamin D Antagonizes Negative Effects of Preeclampsia on Fetal Endothelial Colony Forming Cell Number and Function
Source: PLoS One. 2014 Jun 3;9(6):e98990. doi: 10.1371/journal.pone.0098990 (PMC4044051; doi:10.1371/journal.pone.0098990)
Supplement: Table S1 — Clinical and demographic data for the uncomplicated and preeclamptic pregnancy patients, from whom blood samples were obtained. (DOCX) [file pone.0098990.s002.docx]

**Table S1**. Clinical and demographic data for the uncomplicated and preeclamptic pregnancy patients, from whom blood samples were obtained.

| **Variable** | **Uncomplicated pregnancy** | **Preeclampsia** | **P value** |
| --- | --- | --- | --- |
|  | n=36 | n=30 |  |
| Maternal age (y) | 24.5 ± 4.3 | 26.0 ± 6.5 | 0.47 |
| Gestational age at delivery (wk) | 39.4 ± 1.2 | 37.0 ± 3.1 | <0.01 |
| Multiparous - n (%) | 3 (11%) | 5 (17%) | 0.72 |
| Maternal BMI (kg/m^2^) | 32.8 ± 8.6 | 29.2 ± 6.4 | 0.10 |
| Gestational SBP, pre-delivery (mm Hg) | 123.0 ± 9.2 | 153.0 ± 14.8 | <0.001 |
| Gestational SBP before 20week gestation (mm Hg) | 112.7 ± 6.8 | 117.4 ± 6.5 | <0.04 |
| Gestational DBP, pre-delivery (mm Hg) | 72.0 ± 7.2 | 93.5 ± 8.5 | <0.001 |
| Gestational DBP before 20 week gestation (mm Hg) | 69.0 ± 5.4 | 72.1 ± 5.9 | <0.02 |
| Maternal Race, Black- n (%) | 19 (53%) | 10 (33%) | 0.23 |
| Maternal Race, White – n (%) | 17 (47%) | 19 (63%) | 0.22 |
| Cigarette smoking – n % | 8 (22%) | 1 (3%) | <0.05 |
| Birth weight (g) | 3447 ± 439 | 2598 ± 797 | <0.001 |
| Birth weight percentile | 60.8 ± 27.5 | 29.8 ± 26.3 | <0.001 |
| Birth weight percentile < 10^th^- n (%) | 0 (0%) | 9 (30%) | <0.01 |
| Caesarean delivery- n (%) | 12 (33%) | 12 (40%) | 0.58 |
| Baby gender, male- n (%) | 17 (47%) | 19 (63%) | 0.19 |
| Colony numbers (per 50 million PBMCs) | 2.2 ± 3.6 | 1.0 ± 1.8 | <0.05 |
| Days to first appearance of colonies | 16.8 ± 5.5 | 17.5 ± 4.1 | 0.71 |

BMI, body mass index; DBP, SBP, diastolic and systolic blood pressure; PBMC: peripheral blood mononuclear cells. Data are mean ± SD or number (percent).
