# Supplementary material for: Vitamin D Antagonizes Negative Effects of Preeclampsia on Fetal Endothelial Colony Forming Cell Number and Function
Source: PLoS One. 2014 Jun 3;9(6):e98990. doi: 10.1371/journal.pone.0098990 (PMC4044051; doi:10.1371/journal.pone.0098990)
Supplement: Table S2 — Clinical and demographic data describing the sub-set of patients whose ECFCs were functionally compared. (DOCX) [file pone.0098990.s003.docx]

**Table S2**. Clinical and demographic data describing the sub-set of patients whose ECFCs were functionally compared.

| **Variable** | **Uncomplicated pregnancy** | **Preeclampsia** | **P value** |
| --- | --- | --- | --- |
|  | n=8 | n=8 |  |
| Maternal age (y) | 26.13 ± 5.5 | 24.0 ± 3.5 | 0.37 |
| Gestational age (wk) | 39.8 ± 1.0 | 38.0 ± 3.5 | 0.1 |
| Multiparous- n (%) | 3 (37.5%) | 2 (25%) | 0.5 |
| Maternal pre-pregnancy BMI (kg/m^2^) | 30.8 ± 12.0 | 26.8 ± 4.3 | 1.0 |
| Gestational SBP, pre-delivery (mm Hg) | 123.0 ± 9.4 | 149.4 ± 8.0 | <0.001 |
| Gestational SBP before 20 week gestation (mm Hg) | 113.7 ± 6.3 | 117.4 ± 6.0 | 0.76 |
| Gestational DBP, pre-delivery (mm Hg) | 72.0 ± 9.3 | 93.5 ± 7.8 | 0.003 |
| Gestational DBP before 20 week gestation (mm Hg) | 67.7 ± 3.6 | 73.9 ± 9.0 | 0.66 |
| Birth weight (g) | 3479 ± 448 | 2575 ± 848 | <0.05 |
| Birth weight percentile | 60.9 ± 32.6 | 25.4 ± 27.0 | <0.05 |
| Birth weight percentile < 10^th^- n (%) | 0 (0%) | 3 (37.5%) | 0.2 |
| Caesarean delivery- n (%) | 3 (37.5%) | 3 (37.5%) | 1.0 |
| Race, Black – n (%) | 5 (56%) | 2 (22%) | 0.33 |
| Baby gender, male- n (%) | 3 (37.5%) | 4 (44%) | 0.5 |

BMI, body mass index; DBP, SBP, diastolic and systolic blood pressure (average of last three measurements). Data are given as mean ± SD or number (percentage).
